# Fusing Expert Knowledge with Monitoring Data for Condition Assessment of Railway Welds

**DOI:** 10.3390/s23052672

**Published:** 2023-02-28

**Authors:** Cyprien Hoelzl, Giacomo Arcieri, Lucian Ancu, Stanislaw Banaszak, Aurelia Kollros, Vasilis Dertimanis, Eleni Chatzi

**Affiliations:** 1 Department of Civil, Environmental and Geomatic Engineering, ETH Zürich, Stefano-Franscini Platz 5, 8093 Zürich, Switzerland; 2 Metrology Department, Swiss Federal Railways (SBB), 3018 Bern, Switzerland

**Keywords:** railway infrastructure, condition assessment, Structural Health Monitoring, weld damage, Bayesian Logistic Regression, expert knowledge

## Abstract

Monitoring information can facilitate the condition assessment of railway infrastructure, via delivery of data that is informative on condition. A primary instance of such data is found in Axle Box Accelerations (ABAs), which track the dynamic vehicle/track interaction. Such sensors have been installed on specialized monitoring trains, as well as on in-service On-Board Monitoring (OBM) vehicles across Europe, enabling a continuous assessment of railway track condition. However, ABA measurements come with uncertainties that stem from noise corrupt data and the non-linear rail–wheel contact dynamics, as well as variations in environmental and operational conditions. These uncertainties pose a challenge for the condition assessment of rail welds through existing assessment tools. In this work, we use expert feedback as a complementary information source, which allows the narrowing down of these uncertainties, and, ultimately, refines assessment. Over the past year, with the support of the Swiss Federal Railways (SBB), we have assembled a database of expert evaluations on the condition of rail weld samples that have been diagnosed as critical via ABA monitoring. In this work, we fuse features derived from the ABA data with expert feedback, in order to refine defection of faulty (defect) welds. Three models are employed to this end; Binary Classification and Random Forest (RF) models, as well as a Bayesian Logistic Regression (BLR) scheme. The RF and BLR models proved superior to the Binary Classification model, while the BLR model further delivered a probability of prediction, quantifying the confidence we might attribute to the assigned labels. We explain that the classification task necessarily suffers high uncertainty, which is a result of faulty ground truth labels, and explain the value of continuously tracking the weld condition.

## 1. Introduction

The increasing need for cost reduction and increase in efficiency and safety of railway infrastructure has prompted a surge of data-driven monitoring solutions for optimal management of railway assets [1]. Two essential aspects motivate the need for adoption of automated data-driven track inspection tools [2]: on one hand. the safety of the employees performing visual/on-site field inspections, and, on the other hand, customer comfort and safety [3]. Monitoring-based assessment is achieved by collecting data from specialized diagnostic, as well as from appropriately equipped in-service vehicles, which can provide a network-wide assessment of the railway infrastructure condition [4,5,6] and support preventive maintenance schemes [7,8]. Railway track infrastructure typically consists of continuously welded rails supported by sleepers [9]. Among the critical components of the rail network, welds require particular attention in terms of execution, monitoring, and maintenance [10,11]. By collecting and analyzing data on the condition of such critical infrastructural components, railway operators can better understand the health of their infrastructure and optimize the course of remedial actions [12].

Material imperfections, often originating near welds, grow into more severe faults over time when subjected to repeated stress [13]. These imperfections can include the following: surface defects, which form a broad category of defects caused by factors such as damaged wheels, ballast on the rail surface, or lost goods that induce indentations on the rail; squats, which are defined by the International Union of Railways as a “widening and localized depression of the rail/wheel contact band, accompanied by a dark spot containing cracks with a circular arc or V shape” [14,15]; and cracks, which can appear at the head, web, or foot of the weld and which, although less common, can lead to a broken rail [14,16]. On the railway network operated by the Swiss Federal Railways (SBB), the condition of the track is periodically assessed using data collected from diagnostic measurement vehicles [17]. Diagnostic vehicles are equipped with sensitive and high-precision measurement systems, such as the Rail-Head Imaging System (VCUBE, Mermec Group, Monopoli, Italy) [18] and low-noise piezoelectric accelerometers (Type 4321, Hottinger Brüel & Kjaer, Virum, Denmark) [4]. While such vehicles can only traverse the network at rare pre-planned intervals, On-Board Monitoring (OBM) vehicles, on the other hand, are in-service vehicles equipped with simple and lower-cost sensors, such as microelectromechanical system-based accelerometers, which allow for continuous monitoring [1,19,20,21]. In some implementations, OBM has been scaled down to low-power sensor nodes, further easing installation [22,23,24]. Despite, however, their potential to continuously gather data related to track and vehicle condition across the railway network the use of OBM vehicles has not been generalized yet [4] and, thus, the assessment of critical rail components still largely relies on field inspections [25].

ABA measurements can serve for fault identification and classification, since the dynamic properties of a system often closely reflect its condition [26]. Such a task is accomplished on the basis of two main approaches: model-based or data-driven schemes. Model-based methods, also known as hybrid methods, are physics-based models that are combined with data, in order to accomplish identification tasks, such as the recovery of the rail’s longitudinal level profile from acceleration measurements [27,28]. Model-based schemes enable the identification of the crossing of welds with smooth or degraded surface geometry enabling the identification of potentially faulty welds [29]. Simulations of the crossing of welds on high speed lines were used to estimate the relation between ABA and wheel/rail force and to propose a rail-time health detection method for rail welds [30]. This approach was, however, limited to one type of fault and the results may not fully reflect the varying conditions observed in reality. In the Netherlands, the quality of welds is assessed using gradient approaches on the rail geometry [31]. In such a scenario, limit values on the slope of the rail geometry are derived from a simplified vehicle model with an unsprung wheel mass to estimate the relation between the geometry of the rail, the ABA and the rail/wheel contact force during the crossing of welds [32].

Data-driven methods, on the other hand, are freed from a system model and often rely on transformed representations for extracting features. Typical examples of this class are time–frequency domain analysis methods, such as the Short Time Fourier Transform (STFT), and the Discrete or Continuous Wavelet Transform (DWT/CWT). CWT wavelet coefficients were used by Molodova [33] to classify squats and welds using acceleration data, while, in separate studies, the scale-averaged wavelet power, derived from the CWT coefficients, was applied to identify rail corrugation [34].

Time–frequency domain-based approaches were adopted in further studies for the detection of squat defects [35,36,37,38]. The identification of track quality can also be performed directly on the basis of measured acceleration inputs, which are fed into statistical [39], Machine Learning (ML) or Deep Learning (DL) techniques. Such a principle has been exploited in a number of studies to predict geometric anomalies of the track on the basis of ABA measurements, and, thereby, facilitate the early detection of faults, which might otherwise lead to derailment [40,41].

More recently, Yang et al. [42] demonstrated that both feature-extraction based methods and raw-input based DL methods, such as Convolutional Neural Networks (CNNs), can be applied to detect insulated joints on the basis of acceleration measurements. To further assess the condition of the rail, Tsunashima and Takikawa [43] identified outliers from the CWT spectrograms of ABA. These spectrograms were then analyzed by experts, who noticed that, for small faults, ABA based detection had a higher false positive rate than for larger faults. In their work, Shadfar et al. [44] presented an indicator for condition assessment of rail welds, formulated via coupling of a Fast Fourier Transform (FFT) with Principal Component Analysis (PCA). The authors noted that the performance of this indicator remained to be substantiated in a larger study. A similar approach was proposed by Xiao et al. [45], who combined the wavelet packet decomposition (WPD) with an adaptive synchro-squeezed short time Fourier transform (ASSTFT) to locate damaged welds on a heavy-haul railway line. Previous studies demonstrated the viability of using the Hilbert–Huang Transform (HHT), which is a tool particularly suited for analysis of non-stationary signals, to characterize abnormal vibrations in damaged welds, as a means of monitoring tramway lines [46]. Availability of large datasets containing a range of Environmental and Operational Parameters [47], such as the DR-train dataset [48], allow for the adoption of more complex classifiers. Lasisi and Attoh-Okine [49] predicted the probability of rail fatigue defects by combining several Machine Learning model predictions via Multilayer Stacking Methodology. Their prediction was based on fault logs for the US Class I freight railroad and a set of parameters, such as the track layout, track type and the Million Gross Tonnes (MGT) [49]. Deep Learning approaches have gained more popularity with the appearance of larger datasets enabling the assessment of railway infrastructure with models, such as Convolutional Neural Networks (CNNs) or recurrent neural Networks (RNNs) [40,50,51,52,53]. More recently, approaches for fusing imagery/computer vision with inertial measurements have been proposed. Peng et al. used accelerometer, inclinometer and gyroscopic measurements, combined with image sensors, to quantify track alignment and irregularity and combined this information with visual sensors, but the rail condition (squats and surface defects) was evaluated using the computer vision-based assessment only [54]. Purely data-driven approaches are often limited by the quantity and quality of the training data required to learn a reliable representation of the underlying physical dynamics of the vehicle/track system.

In previous works of the authoring team [51], welds, surface defects, squats, and insulated joints were successfully classified using a dataset of over 200,000 instances, via the use of machine learning techniques, namely Random Forests (RFs) and CNN [51]. This was initial work towards the automated classification of essential rail elements on the basis of ABA data. In subsequent work [55], this approach was extended, via the use of an outlier-based detection scheme, to identify potentially faulty welds. The outcome of this investigation was adopted in practice by the SBB in a Proof-of-Concept study, where the suspected faulty instances were delivered to experts for subsequent assessment. The resulting expert-labeled dataset of healthy and defective welds formed the initial dataset, which was exploited in this study. This dataset was, then, further complemented with the welds whose condition labels stemmed from the classical track inspection process. For the SBB, such a process is logged in the form of entries in a so-called condition monitoring database ZMON (ZustandsMONitoring—condition monitoring). As highlighted in Table 1, several studies have demonstrated the feasibility of the use of ABAs for identification of squats, welds and insulated joints, usually on a small selection of samples. No study so far has proposed a component-specific and large-scale assessment, which attempts to fuse OBM indicators (acceleration-based ratings) with expert feedback. This study focused on the treatment of welds; a common component of the railway network, whose assessment is critical for alleviating faults that can cause increased costs or compromise safety [17].

**Figure 1:**
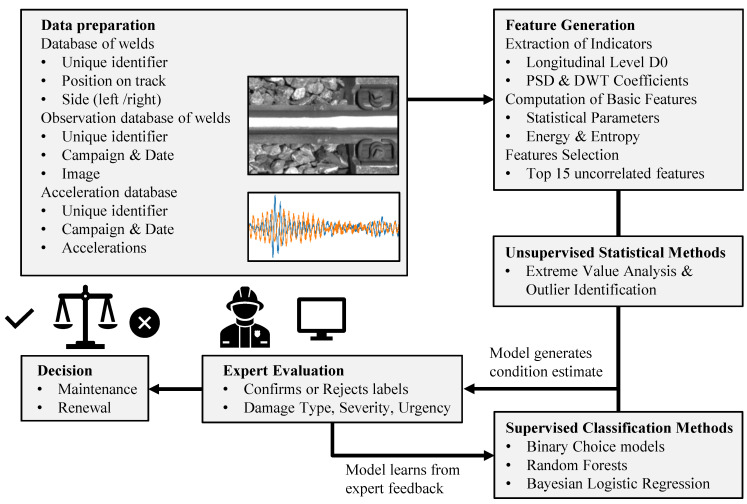
Flowchart summarizing the proposed methodology applied for automated weld defect detection and classification from ABA data. The accelerations and rail-head images are continuously collected by the diagnostic vehicle of the SBB (gDFZ) and extracted at the location of welds. Outlier welds, that are statistically identified from the features extracted from the ABA data, are subsequently delivered for expert assessment with complementary input of the available image data. The expert assessment is then used to retrain the models using supervised ML algorithms. Finally, the experts use the improved model to guide their decisions on maintenance and renewal.

This paper addresses this research gap by proposing a method based on ABA data for automatic defect detection on welds. The proposed methodology, which builds on a two-step procedure to assess weld condition, is illustrated in Figure 1. First, outlier welds are identified using Extreme Value Analysis (EVA) applied to the ABA indicators, that are extracted from the vehicle track interaction measurement system of the diagnostic vehicle of SBB. The images of these potentially faulty welds, extracted from VCUBE, are, then, submitted to experts for visual assessment, on the basis of which a dataset of expert-based condition labels is generated. The feedback from the expert evaluation round is exploited as an extension of the work in [55] to develop a classification system to distinguish between healthy and defective welds based on ABA indicators. The following three alternative analysis methods are examined here for the supervised approach: a pair of more conventional assessment schemes relying on (i) Binary Choice classification and (ii) Random Forests, and (iii) a herein proposed approach which adopts a Bayesian Logistic Regression scheme that capitalizes on availability of expert input. The novelty of this approach lies in the integration of human expertise with statistical assessments on ABA indicators, which allows for a more effective and efficient evaluation of welds. By integrating expert feedback into the ABA-based condition assessment, we achieve a significant advance in the field of condition assessment of railway assets which has the potential to improve the accuracy, consistency, efficiency, and cost-effectiveness of the inspection process. This PoC constitutes a first step towards actionable integration of acceleration-based infrastructure condition assessment into the monitoring process of railway operators.

## 2. Description of the Measurement Data

To ensure a representative dataset, the welds assessed in this work were selected from rail-track portions throughout the Swiss network, namely the west, south, center and east regions. The selected railway tracks were amongst those that were regularly inspected by the diagnostic vehicle (gDFZ) and were further accessible for on–site inspections by experts, when required. The gDFZ was equipped with vertical and lateral ABA on the left and the right side of the front (axle 1) and rear axle (axle 4) of the vehicle. The sensor range was ±100 g and the sampling rate was $F_{s}=24$ kHz. The naming convention of the associated sensors, complying with railway–specific standards, was $D_{A\;S}$, with the related letter-entries explained in Table 2.

**Figure 2:**
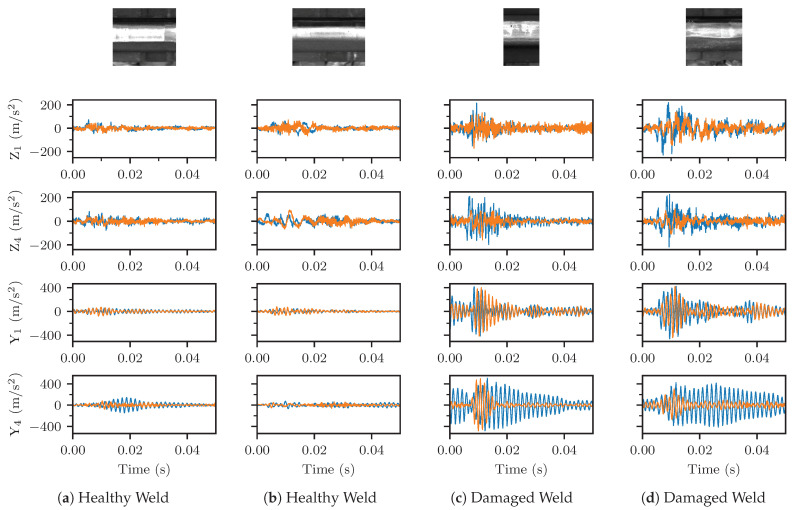
Illustration (VCUBE) and ABA time series for two healthy (**a**,**b**) and two damaged (**c**,**d**) welds. Each figure shows the left (orange) and right (blue) ABA time series, respectively, for the naming convention defined in Table 2.

The location of welds on the railway network was obtained from an automated detection algorithm developed at SBB. It relies on images from VCUBE and determines rail components, such as welds, insulated joints and surface defects [17]. Data obtained between June, 2021, and June, 2022, was, herein, used to generate a weld database containing around 25,000 unique welds. While the automated rail-head inspection system does not always detect all existing welds, the repeated measurements increase the probability of individual weld detection. Approximately 10 vehicle runs were conducted within this one year period, implying that the majority of welds were repeatedly measured in this interval, allowing for tracking of their condition over time. The resulting collected ABA samples are illustrated in Figure 2, which shows the VCUBE images and the measured ABA for two healthy (Figure 2a,b) and two damaged weld (Figure 2c,d) cases. The damaged welds showed higher ABA on all channels compared to the healthy welds. Figure 2 shows the left and right ABA time series for the vertical ABA on the leading axle Z_1_, the vertical ABA on the trailing axle Z_4_, the lateral ABA on the leading axle Y_1_ and the lateral ABA on the trailing axle Y_4_. The ABA data samples corresponding to welds recognized by VCUBE were processed accordingly, as described in Section 3.1.

## 3. Methodological Approach

We propose a framework which exploits labeling of rail infrastructure defects on the basis of expert evaluation on outliers in ABA data, acquired from instrumented trains. An automated classification framework for weld condition monitoring is, thus, established, that can be applied to newly acquired measurement data, potentially from in-service trains.

To achieve this goal, we first extracted a range of features from the ABAs of a properly instrumented diagnostic vehicle. These were calculated over the whole data range (one year period). Following a statistical characterization of the features, extreme value analysis was performed and outliers were identified. The latter were submitted to experts, and a large database of fault characteristics was generated. The expert-feedback was finally incorporated into a classification framework, which scored the quality of welds, based on the coupling of ABA features with expert-feedback.

### 3.1. Feature Extraction

The efficient identification and classification of outliers calls for the extraction of representative features from the ABA time series. A critical requirement for an interpretable anomaly detector is the computation of features that provide an intuitive and comprehensive illustration of the state of the assets from the measured time series [57].

The fundamental quantities, on which features were extracted, are listed in Table 3 and further explained in Section 3.2. The signal length that was used to compute the features was determined on the basis of two criteria. First, the signal length should be sufficiently long to contain the analyzed wavelengths and/or frequencies. For example, a signal length of 5 m around the weld was chosen for the longitudinal level D0 (see Section 3.2 below), since the filtered wavelengths were up to 3 m. The second criterion stems from uncertainty in vehicle position when crossing the welds. This positional uncertainty was tackled by choosing a rather large signal length around the defect (0.625 s for the DWT features or around 2–3 m for all other features).

The required sparse representation of essential features was achieved by computing statistical indicators on the basis of the extracted features [58]. These included minimum, maximum, mean value, standard deviation, and quantiles, as well as higher statistical moments, such as skewness and kurtosis. Statistics were computed for each quantity of Table 3, and for every channel in the vertical *Z* and lateral *Y* direction, where applicable. As multiple vehicle axles were equipped with sensors, the statistical indicators were also aggregated between the four sensor locations using the mean, minimum and maximum of the single channel statistics.

For example, one can consider the maximum vertical acceleration for each of the ABA sensors separately, or the maximum (or mean of the maximum) vertical acceleration over the four sensor locations, in order to form aggregated summary statistics. Such statistics capture the variability in contact conditions mainly arising from defect size, which, for small defects, can lead to greater variance in response between vehicle axles.

Operational parameters, such as the vehicle speed, were additionally included in our assessment framework, since they retain a non-negligible influence on the axle response.

### 3.2. Time Series Analysis

During the crossing of a damaged weld, increased vibration levels were observed in the vertical and lateral ABAs. In particular, impacts on the axle caused high amplitudes in the lateral and vertical vibration of the rail–axle system, whose first resonant frequencies lay, approximately, around 660 Hz and 1 kHz, respectively [59,60].

**Figure 3:**
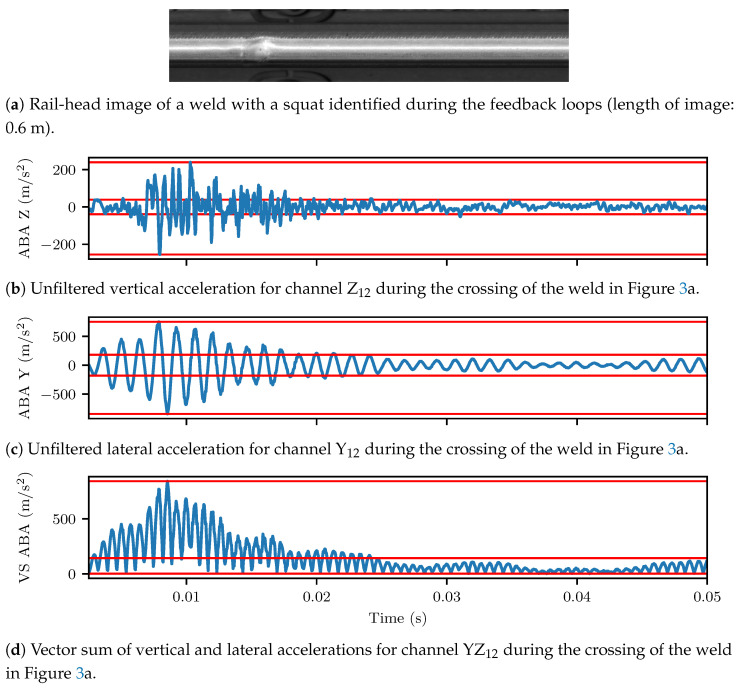
Feature extraction from the vertical and lateral ABAs. The vertical and lateral ABAs were combined using the vector sum. Where applicable, the continuous red horizontal lines highlight the maximum, minimum and standard deviation of the time series.

Indicatively (in the sequel, all signals were mean value subtracted prior to any processing applied), vertical peak accelerations of up to 200 m/s^2^ and lateral peak accelerations of up to 600 m/s^2^ were noted during the crossing of the damaged weld shown in Figure 3a for both the vertical (Figure 3b) and lateral (Figure 3c) ABAs. The vertical ($s_{Z}$) and the lateral ($s_{Y}$) acceleration components were, herein, further combined, by computing the vector sum of both signals, i.e.,

(1) $$s_{\mathrm{vector}\;\mathrm{sum},YZ}={\left(s_{Y}^{2}+s_{Z}^{2}\right)}^{\frac{1}{2}}$$

where *s* is the time series of the respective ABA channel. Figure 3d shows the vector sum computed from the lateral and vertical ABA of the damaged weld.

**Figure 4:**
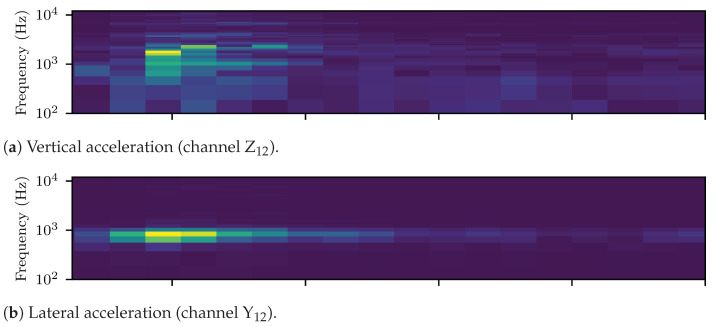
Spectrograms (Welch’s method with $N_{\mathrm{FFT}}=128$, Hanning window and 50% overlap) of the high pass filtered (100 Hz cutoff frequency) vertical and lateral ABAs of Figure 3b,c.

When investigating high frequency vibrations, the signals of Figure 3b,c were zero-phase, high-pass filtered using a 6th order digital Butteworth filter at 100 Hz cutoff frequency. Figure 4a,b display the spectrograms (Welch’s method with $N_{\mathrm{FFT}}=128$, Hanning window and 50% overlap) of the filtered vertical and lateral ABAs, respectively. The previously identified pinned–pinned resonant frequencies of the rail/axle system lay around 700 Hz, laterally, and 1 kHz, vertically, for the standard UIC60 rail [59,60]. These frequencies are identified from Figure 4a,b. The rail–wheel system was less stiff and less damped laterally, which resulted in higher vibration amplitudes compared to the vertical wheel-set response. Moreover, critical vibration response of the damaged weld was observed for frequencies up to 12 kHz. For subsequent analysis of the ABA data, a set of empirical frequency bands was, thus, formulated. These were the following: (i) 200–500 Hz, (ii) 500–800 Hz, (iii) 800 Hz–2 kHz, (iv) 2–4 kHz; and (v) 4–11 kHz. The associated signals, which form the BP ABA entry of Table 3, resulted from corresponding filtering of the original ABA, using the same zero phase Butterworth filter as before, applied in band pass mode at the selected ranges.

Similar results for the frequency content of the ABA were extracted by applying the Discrete Wavelet Transform (DWT) [61]. The DWT was, herein, implemented using the Haar mother wavelet and successive filtering operations with two FIR filters: a low-pass filter $h_{\phi }$ and a high-pass filter $h_{\psi }$. The associated approximation $W_{\phi }\left(j,k\right)$ and detail $W_{\psi }\left(j,k\right)$ coefficients of the *j*-th scale were computed by convolution [62]

(2) $$\begin{array}{cc} W_{\phi }\left(j,k\right) & =h_{\phi }\left(-n\right)*W_{\phi }{\left(j+1,n\right)|}_{n=2k,k\geq 0} \end{array}$$

(3) $$\begin{array}{cc} W_{\psi }\left(j,k\right) & =h_{\psi }\left(-n\right)*W_{\phi }{\left(j+1,n\right)|}_{n=2k,k\geq 0} \end{array}$$

**Figure 5:**
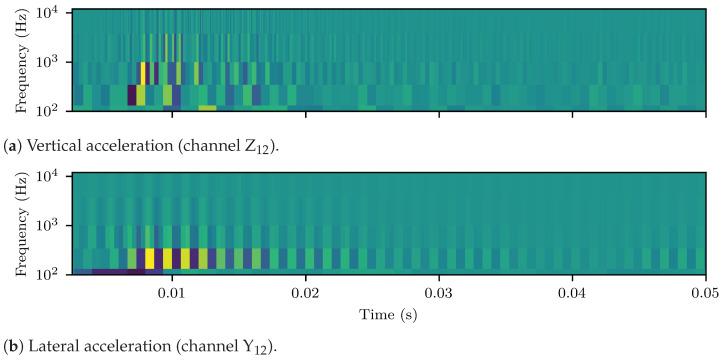
DWT (via Haar wavelet) of the high pass filtered (100 Hz cutoff frequency) vertical and lateral ABAs of Figure 3b,c.

Figure 5a,b illustrate the detail coefficients computed for the high pass filtered vertical and lateral ABA time–series of the weld in Figure 3a. The same insight, as in the case of the spectrogram, was obtained for the effective frequency bands of the signals.

Geometric features, such as the longitudinal level, or the lateral alignment, reflect the vertical or lateral smoothness of the rail [63]. These quantities are defined for several wavelength ranges by the railway norm EN13848-1 [64], namely D0 (wavelength 1–3 m), D1 (wavelength 3–25 m) and D2 (wavelength 25–70 m). Using integration and filtering techniques on ABAs, one can obtain robust, speed independent and repeatable indicators corresponding to the longitudinal level and lateral axle displacement [1,4,27,65].

When estimating the longitudinal levels, the ABA signals were initially zero phase, band-pass filtered (0.5 Hz to 75 Hz, 6th order digital Butterworth filter) and resampled at 150 Hz. The cumulative trapezoidal numerical integration method was accordingly applied to yield the double integrated vertical and lateral displacements. Drifts stemming from the integration process were removed by applying a 6th order Butterworth high-pass filter with a cutoff frequency of 0.5 Hz, which corresponded to the minimum frequency response of the sensor. The resulting displacement signals were then transformed from time series to space series, using a wavelength rate of 25 cm. Finally, appropriate band-pass filters were applied, to obtain the longitudinal levels and lateral displacements D0, D1 and D2. This approach for longitudinal level recovery has been successfully applied at the SBB [4] and the German Railways [1].

**Figure 6:**
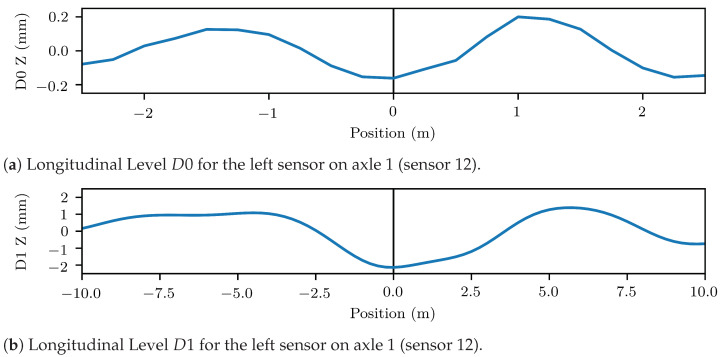
Longitudinal Levels D0 & D1 for the time series of Figure 3b. One can observe local settlements occured for both the 1–3 m range of D0 and the 3–25 m range of *D*1.

The longitudinal levels D0 and D1 computed from the vertical ABA are illustrated in Figure 6a,b, respectively. These correspond to the level during the crossing of the damaged weld of Figure 3a. The plots revealed settlements, which were commonly observed at the location of the weld in both the short wavelengths under 3 m and the medium wavelengths between 3–25 m. These localized track settlements are the result of the repeated impacts of the vehicle wheels on the damaged weld [66]. The settlements occurring in the wavelength range of 1–25 m could be attributed to further causes, such as changes in the track stiffness and substructure condition [67], thus, forming a less robust indicator of rail and weld condition.

### 3.3. Extreme Value Analysis for Outlier Identification and Expert Labeling

A limitation of supervised machine learning approaches is the limited availability of labels, as well as their quality, since these are often linked to subjective and, therefore, biased assessment. For the dataset we were handling herein, image labels could be extracted. However, the automatic image labeling algorithm of the SBB does not currently output the condition of welds; a task which would be non trivial to effectuate. Capitalizing on the availability of the collected ABA signals, we here adopted an unsupervised scheme, applied directly to raw time history ABA signals, or to the aforementioned features, to detect abnormal, and potentially faulty, welds.

Extreme Value Analysis (EVA) is a statistical technique that is used to analyze the likelihood and impact of the occurrence of extreme events, such as floods, hurricanes, and earthquakes. In practice, EVA often relies on the use of extreme value distributions, such as the Gumbel, Fréchet, and Weibull distributions [68], or even empirical distributions [69], which are formed on the basis of available data. The cumulative density function of an empirical distribution is formulated as [70]

(4) $${\tilde{f}}_{\mathrm{ED}}\left(t\right)=\frac{i}{n+1}\;\mathrm{for}\;x_{i}\leq x<x_{i+1}$$

where $\left\{x_{1},\ldots ,x_{n}\right\}$ is an ordered sample of *n* independent observations. ${\tilde{f}}_{\mathrm{ED}}$ is an estimate of the true probability distribution *f*, and should be in reasonable agreement with the candidate model (e.g., $f_{\mathrm{Gumbel}}$), provided the candidate model is an adequate estimate of *f* [70]. The Gumbel distribution is commonly applied for modeling the behavior of extreme events and may have been an alternative to the empirical distribution.

The non-parametric empirical distribution is fitted to the data and is subsequently used to estimate the probability of specific outlier level occurrences. The probability estimates resulting from the empirical distribution enable the estimation of the likelihood of the occurrence of a specific level of an ABA-based feature on a component. The computation of the likelihood of occurrence of an extreme value enables the assessment of the potential damage of a defect (outlier) weld. This further requires the definition of threshold of damage levels for ABA-extracted features. EVA was here adopted as the first step of our proposed assessment framework, in order to identify outlier welds that could be subsequently labeled by experts, who were shown rail-head and track inspection system images extracted from the diagnostic vehicle. The number of samples that could be evaluated by the experts was limited, which must be taken into consideration when setting thresholds for outliers. Expert-based labeling requires significant time, as each sample is checked individually. Therefore, EVA was used in the first step to identify and forward only suspected defect welds for cross-checking and labeling. Section 4.1 details the practical implementation of the expert labeling process, the selected thresholds on the ABA features and the results of the expert evaluation. The expert feedback resulted in a labeled weld condition dataset, which, in turn, enabled the establishment of an automated classification scheme, as described in Section 3.4.

### 3.4. Expert-Informed Classification Models

The derived expert labels were exploited for automated weld damage classification, via the use of machine learning classification tools. The binary approach used here, to distinguish between healthy and defective, stemmed primarily from the fact that the magnitude of a defect is not a clearly defined criterion among experts nowadays.

In the most simple scenario, Binary Choice (BC) models assign a choice between two discrete alternatives (in this case defective or healthy) on the basis of a classification rule depending on one variable *x*. This was used here as an approach to an one-class classification between a defective or healthy weld, in the sense of what expert judgment tried to offer. However, it has to be emphasized that this is not entirely consistent with the goal of continuous monitoring. In reality, the task of characterization of defect welds should also take the damage severity into account, which, however, is a label that is currently missing. A characterization on the basis of severity of the defect is valuable and can be provided via ABA data, which can pick up the initiation and evolution of a defect. Expert labels, on the other hand, tend to only acknowledge quite progressed defects. Section 4.3 indicates how such tracking can be accomplished on the basis of ABA measurements.

Returning to one-class classification, when considering a BC model, the threshold which defines the limit for the decision on a healthy or defective weld can be defined as

(5) $$P_{\mathrm{BC}}\left(y|x\right)=\left\{\begin{array}{ll} 1 & x>\gamma \\ 0 & x\leq \gamma \end{array}\right.$$

where *y* is the label from the expert assessment, *x* is the statistical indicator and γ is the decision threshold. For indicator values *x* larger than the decision threshold, the sample is assumed to be defective.

**Figure 7:**
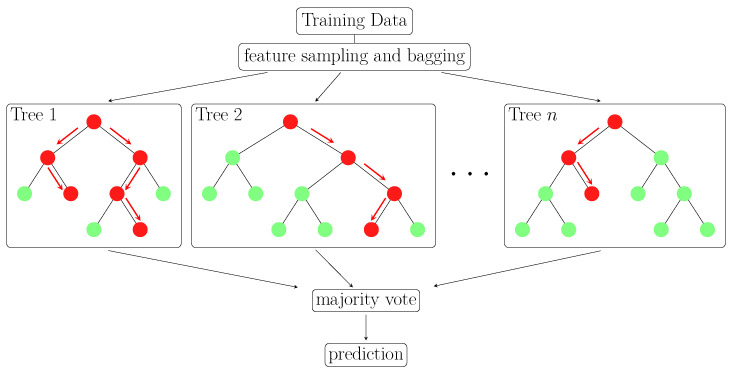
Conceptual illustration of a Random Forest composed of *n* trees for a two class classification problem. Cross-Validated Grid Search was used to determine the optimal hyperparameters of the RF as having a minimum number of samples per split $n_{split}=20$, a minimum number of samples per leaf $n_{leaf}=10$, a maximum depth $n_{depth}=10$, and a number of estimators *n* = 100.

Beyond mere classification, however, alternate models relying on a graph structure, such as Decision Trees (DTs), or their ensembles, Random Forests (RFs), can further point to a root cause analysis path [71,72]. In other words, they can reveal variable configurations which lead to a specific outcome. DTs are a graph structure, in which each internal node denotes the outcome of a test on an attribute, each branch denotes the result of the test, and each leaf node (end node) denotes a class label. The paths from root to leaf represent the classification rules. Figure 7 conceptually illustrates three DTs, which are combined into one RF. RFs are ensemble models that aggregate several DTs to achieve a more robust prediction than an individual DT. The methodology proposed here employed RFs to classify the class label, based on the features extracted from the ABA signals. More formally, given a set of *N* decision trees $\left\{T_{1},T_{2},\ldots ,T_{N}\right\}$ in the forest, the prediction of the RF was achieved by aggregating the prediction of the individual DTs of the class label *y* for a set of essential indicators x = $\left\{x_{1},x_{2},\ldots ,x_{k}\right\}$

(6) $$P_{\mathrm{RF}}\left(y\right)=\sum_{i=1}^{N}\frac{T_{i}\left(x\right)}{N}$$

Many models, including RF, benefit from a limited collinearity of the variables [73] by manually discarding variables using the Pearson correlation or combining them via Principal Component Analysis [74]. The essential indicators selected for the evaluation of welds via RF and BLR, summarized in Figure 4, were the ones which had the highest F1-score in the univariate BC model scenario and which had a Pearson correlation of less than 0.8 to the other indicators.

Figure 7 conceptually illustrates the structure of an RF. RFs include a set of hyperparameters, such as the number of estimators, the minimum number of samples per split, the maximum depth, and the minimum samples per leaf. The optimal parameters of the RF were here estimated using a Cross-Validated Grid Search [75]. The optimal set of hyperparameters identified by the Cross-Validated Grid Search for the Random Forest was a minimum number of samples per split $n_{split}=20$, a minimum number of samples per leaf $n_{leaf}=10$, a maximum depth $n_{depth}=10$, and a number of estimators *N* = 100. The RF model proposed here relied on the implementation of scikit-learn [75], where the Shannon entropy loss $H(X_{m})$ was used as the tree node splitting criterion [76]

(7) $$H\left(X_{m}\right)=-p_{\mathrm{healthy},m}log\left(p_{\mathrm{healthy},m}\right)-p_{\mathrm{defect},m}log\left(p_{\mathrm{defect},m}\right)$$

where $p_{n,m}$ is the proportion of observations of each class *n* at a given node *m*. The Shannon entropy quantifies the expected uncertainty inherent in the possible outcomes of a discrete random variable; in other words, it quantifies the impurity in a group of observations. Thus, for each node, the tree splitting criterion was set such that the entropy loss $H(X_{m})$ was minimized for the data $X_{m}$ at node *m*. Each decision tree was obtained by recursively partitioning the feature space using the previously defined entropy loss function until the constraints defined by the hyperparameters (e.g., tree depth) were reached. The RF was obtained by initializing *N* decision trees with a split composed of a random set of features and random training samples. The aggregation of several DTs in a RF resulted in a more robust prediction compared to single DTs.

An alternative tool for automated classification lies in the adoption of Bayesian statistical models. In this work, we proposed a Bayesian Logistic Regression (BLR) model. Given our set of features, a logistic regression modeled the probability of the weld being damaged as

(8) $$P_{\mathrm{BLR}}\left(y|\alpha ,\beta_{speed},\beta_{1},\ldots ,\beta_{15}\right)=\sigma \left(\alpha +\beta_{speed}x_{speed}+\sum_{i=1}^{15}\beta_{i}x_{i}\right)$$

where $\sigma \left(t\right)=\frac{1}{1+e^{-t}}$ is the logistic function, $x_{speed}$ and $x_{i}$ form our set of predictor variables, and α, $\beta_{speed}$ and $\beta_{i}$ are the parameters of the model to be estimated for the linear transformation of the feature vector (see [77] for an in-depth discussion of the model). The set of essential indicators $x_{i}$ and $x_{speed}$ corresponding to the BLR parameters $\beta_{i}$ and $\beta_{speed}$ are summarized in Table 4. In order to determine the parameters of the model, for which a closed form solution is not generally available, a maximum likelihood approach can be used. However, this comes at the cost of certain drawbacks. First, a maximum likelihood approach is prone to over-fitting. Second, a set of possible solutions is generally available, but this approach determines a single solution, which highly depends on the adopted optimization algorithm. Furthermore, the assigned labels are often noisy, as is typical in real-world measurements, and as a consequence of the aforementioned subjectivity of the expert assessment. A maximum likelihood approach cannot, however, provide an indication of inherent uncertainty. For these reasons, in this work we adopted a Bayesian estimation of the logistic regression.

**Figure 8:**
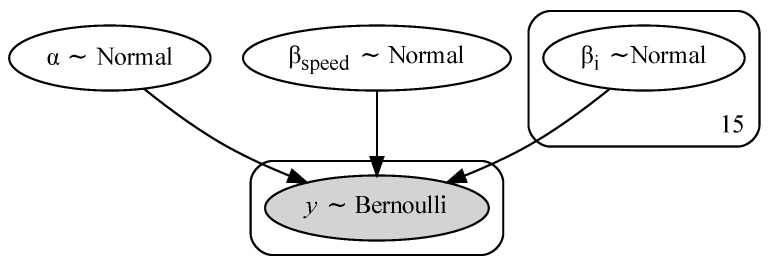
Bayesian Logistic Regression model, where the coefficients α, $\beta_{speed}$, $\beta_{i}$ were assigned a normally distributed prior. The prediction *y* followed a Bernoulli distribution.

A BLR model [77] solves the aforementioned issues in the following way: (i) reducing the risk of over-fitting thanks to the regularization of the priors; (ii) producing a distribution of possible model solutions under the model assumptions (i.e., the priors); (iii) providing a more reliable indication of the predictive uncertainty. Again, exact Bayesian inference of the logistic regression is intractable and approximate methods are generally used. We, here, estimated the BLR model by Markov Chain Monte Carlo (MCMC) sampling, exploiting the No-U-Turn Sampler (NUTS) algorithm [78]. The BLR model was implemented with the probabilistic programming Python package PyMC4, which allows for flexible specification of Bayesian statistical models [79].The model parameters were assigned a Gaussian prior N(0,1), while the labels were modeled through a Bernoulli likelihood. Four chains were used in the MCMC inference, with 2000 sample draws and 10,000 tuning samples per chain. A draw refers to a collected sample generated from the posterior distribution of the MCMC inference, while tuning samples are generated before starting to collect posterior samples and are used to tune the sampling algorithm by adjusting the step size of the updated distribution, as well as to ensure the convergence of the chains. The graphical model of the implemented BLR is displayed in Figure 8.

An expert-in-the-loop approach requires the expert feedback to adjust the anomaly detection scheme, such that the outlier detection classifier or decision threshold are more in tune with the expert’s understanding of anomalies. Here, the feedback was incorporated using the labels generated by the expert assessment during the training stage. The process used for applying the expert labels in the classification process is illustrated in Figure 1, where the expert labels were essentially used to improve the model performance in the supervised classification framework.

## 4. Results and Discussion

In this section, we elaborate on the results from the expert-based evaluation of outlier welds and their influence on classification of their condition. We compared performance on identification of faulty welds for the three schemes outlined above, namely the BC, RF and BLR model.

### 4.1. Expert-Based Evaluation of Outlier Welds

#### 4.1.1. Definition of Capacity-Based Thresholds

Semi-supervised approaches require the definition of suitable outlier metrics. We specified these, here, on the basis of the two main defect types that are encountered for welds, namely geometric defects or surface defects/squats. A third category of defects can be attributed to internal effects, such as cracks, which are, however, not visible, and would not be possible to assess through expert visual inspection. These may, however, be labeled through non-destructive evaluation, which is logged to the ZMON database, as explained in Section 4.1.3. In this subsection, we restricted evaluation tp the visual inspection of experts, for which the first two defect instances were relevant. Geometric defects are linked to decreased longitudinal level values ($D0_{Z,min}$), which point to a degraded weld geometry [31]. On the other hand, surface defects are linked to peaks in acceleration and energy values [80]. Therefore, we used the maximum vertical acceleration (${\mathrm{ABA}}_{Z,max}$) and the longitudinal level ($D0_{Z,min}$) as the main metrics for selection of outliers.

In order to define thresholds for outlier selection, we employed EVA, as described in Section 3.3. To this end, we fitted an empirical distribution to the aggregated values of $D0_{Z,min}$ and ${\mathrm{ABA}}_{Z,max}$, collected on records from all available weld samples. Two thresholds were defined, associated with the 98-th and 95-th percentiles of the fitted EDs, corresponding to strong and weak outliers, respectively. The choice of percentile for the strong outlier case was carried out so as to include instances of weld defects that were discovered in the field through visual inspection, but which had not been picked up by the automated image-based detection system of the diagnostic vehicle (VCUBE), which was considered to be a rare incident. Strong outliers were defined only on the basis of maximum vertical ABAs, as we suspected this indicator to be more directly related to the weld defects. The weak outlier definition combined information from both the maximum vertical ABA and the longitudinal level D0, as we suspected that the longitudinal level plays a role, albeit secondary, in the degradation process of welds. The definition of the sets of strong $S_{s}$ and weak $S_{w}$ outliers, given an observation *k*, was formulated as follows:

(9) $$\begin{array}{cc} S_{s}=\{k|\;O_{s}= & \frac{{\mathrm{ABA}}_{Z,max,k}}{q_{98\%}\left({\mathrm{ABA}}_{Z,max}\right)}>1\}\\ S_{w}=\{k|\;O_{w}= & {\left(\frac{D0_{Z,min,k}^{2}}{q_{95\%}{\left(D0_{Z,min}\right)}^{2}}+\frac{{\mathrm{ABA}}_{Z,max,k}^{2}}{q_{95\%}{\left({\mathrm{ABA}}_{Z,max}\right)}^{2}}\right)}^{0.5}>1\;\& \end{array}$$

(10) $$\begin{array}{cc} \;O_{s}= & \frac{{\mathrm{ABA}}_{Z,max,k}}{q_{98\%}\left({\mathrm{ABA}}_{Z,max}\right)}<1\} \end{array}$$

where ${\mathrm{ABA}}_{Z,max,k}$ is the maximum vertical ABA for the *k*-th observation and $D0_{Z,min,k}$ is the minimum longitudinal level in proximity of the weld for observation *k*.

An amount of 100 outliers per region and per trimester (evaluation round) was deemed as realistic, to be checked by the assigned experts. The described process resulted in a total of 195 strong outliers, during the first expert evaluation round. However, the evaluation of weak outliers resulted in more samples than could feasibly be evaluated by the experts. Thus, a random selection amongst the weak outlier set was carried out to reach a total of 100 strong and weak outliers per region and per evaluation round (trimester).

**Figure 9:**
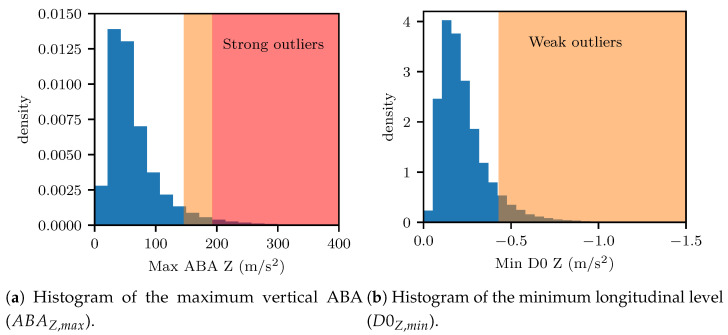
Distribution of the maximum vertical ABA (${\mathrm{ABA}}_{Z,max}$) and the minimum longitudinal level ($D0_{Z,min}$).The cutoff thresholds were defined on the basis of the 98-th percentile of the empirical distribution of ${\mathrm{ABA}}_{Z,max}$ for the strong outlier region (highlighted in red), per Equation (9), and on the basis of the 95-th percentiles of the minimum longitudinal level $D0_{Z,min}$ and ${\mathrm{ABA}}_{Z,max}$, as formulated in Equation (10), for the weak outlier region (highlighted in orange), respectively.

Figure 9 illustrates the distributions of the selected outlier metrics, namely, the maximum vertical acceleration (${\mathrm{ABA}}_{Z,max}$) and the minimum longitudinal level ($D0_{Z,min}$) values. Furthermore, the defined outlier regions are highlighted in Figure 9. For most welds, it was observed that the peak ABA lay under 100 m/s^2^ and that the minimal longitudinal level was lower than 0.3 mm in absolute terms. The defined outlier metrics were used in the next section to deliver samples for expert assessment.

#### 4.1.2. Expert Assessment

In the proposed framework, the outliers defined from the EVA analysis in Section 4.1.1, were forwarded to the experts for cross-check and labeling. The process illustrated in Figure 1 was conducted with actual rail monitoring data, as part of a PoC project, enabling a feedback loop between the experts, asset managers and researchers. Outlier welds, as defined in Section 4.1.1, were labeled by experts on the basis of the black & white image feedback offered by rail-head images acquired from the diagnostic vehicle and the VCUBE system. Over the period of one year, four feedback loops were performed, during which a total of 1727 outlier welds were delivered to the experts for evaluation. The thresholds defined prior to the first iteration [55] were kept identical to track the expert evaluations on critical welds over time. The welds submitted for evaluation over all feedback cycles in this study were composed of 911 samples that featured strong outlier scores, and a selection of 816 weak outlier samples. The weak outlier selection corresponded to random samples selected in order to achieve 100 samples per region and per expert evaluation round. The condition of the welds was then assessed based on the criteria defined in the deviation catalog of SBB [81]. Deviations in this catalog are for instance welds featuring a squat, a surface defect or faults in the geometry.

A two stage identification process was carried out. In the first step, the experts were asked to identify if the outlier corresponded to a weld. After four evaluation rounds, 132 samples were not evaluated due to the time-constrains of the inspectors. In 113 cases, the image-based system recognized other faults as welds and for 1491 samples the experts recognized a weld in the image, delivering important information on the performance of the rail-head image-based weld detection (see also Figure 11). In the second step, the experts visually assessed the condition of the welds using the VCUBE images. The results of the expert evaluation, upon completion of the four feedback loops, are summarized in Table 5. Around 12% of the strong outliers and 6% of the weak outliers were labeled as defective by the in-office experts.

**Figure 10:**
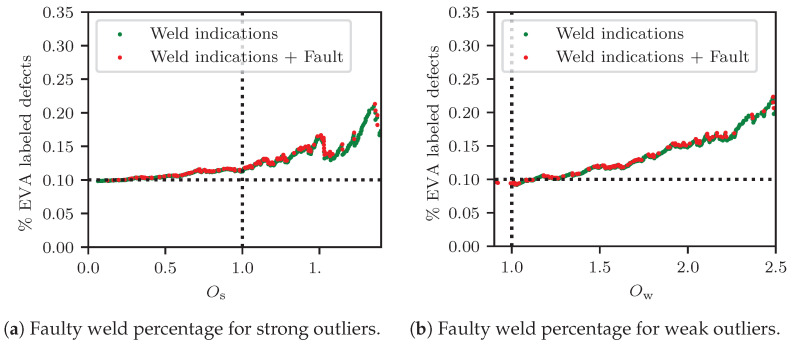
Percentage of damaged welds in the samples evaluated by experts after four evaluation rounds, given outlier scores that were higher than the prescribed thresholds. At higher outlier scores, 22% of the ABA-based outliers were assigned a faulty label by the experts.

Figure 10 illustrates the percentage of defect welds versus the values of the corresponding scores for strong and weak outlier sets defined in Equations (9) and (10). The vertical dashed line indicates the threshold between strong and weak outlier regions. The dotted horizontal line offers a visual indicator for regions where the number of defects in the delivered sample was higher than 10%. The green markers indicate the welds that were evaluated as healthy on the basis of expert visual assessment, while the red markers show the defective instances. It is evident that, under increasing outlier scores, the ratio of welds that were assigned a defective label versus the complete set of ABA-defined outliers rose from 10% to 22%. At higher outlier scores, a variability was noted in terms of the outlier score due to the fewer remaining samples, resulting in decreased statistical significance. It needs to be here noted, however, that ABA information can deliver defects in their initiation or formation, which may not be deemed as faults via the expert visual inspection. Heavier weld faults are most commonly attributed to the presence of squats or another distortion in the welding, rather than tied to geometric faults. The outlier scores were, here, formulated so that samples in the weak outlier set included the instances that corresponded to anomalies in the geometry. It should here be noted that, as the evaluation criteria were mainly visually-based, the expert evaluation could not thoroughly capture geometric irregularities, which were smoother (influencing larger wavelengths than a local squat). In addition, the evaluation was affected by inspector bias (see also Figure 14). The challenges resulting from the uncertainty in the ground truth are further discussed in Section 4.2.

#### 4.1.3. Fusion of Data from the Standard Condition Monitoring Database (ZMON)

As part of the standard evaluation procedure, welds that are inspected and deemed as defective by experts are recorded in the ZMON (ZustandsMONitoring—condition monitoring) database. This is a condition logging database containing faults stemming from the following: (i) visual inspections; (ii) more specialized Non-Destructive Evaluations, such as ultrasonic inspections conducted by a system mounted on a dedicated diagnostic vehicle; (iii) automated track inspections of rail faults by means of the VCUBE system, mounted on a specialized diagnostic vehicle. Faulty welds that are picked up by the ultrasonic inspection vehicle are verified by on-site measurements using a handheld device. Furthermore, faults that are picked up by both the automated track inspection system (VCUBE) and visual inspection need to be first validated by experts in the office, prior to being added to ZMON. This database serves for efficient planning of maintenance and renewal actions.

**Figure 11:**
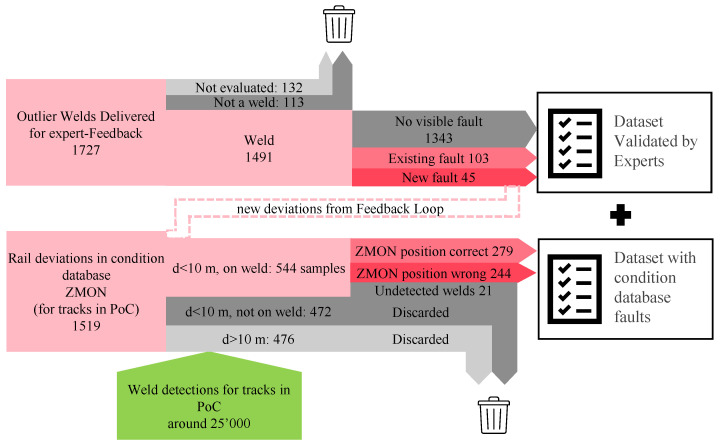
Process for generating weld condition labels from the ZMON condition database on the basis of expert feedback conducted on the ABA-derived outliers, as well as through the standard inspection and evaluation processes, which include visual (on-site), VCUBE-based, and ultrasonic-based track inspections.

During the expert evaluation process that was executed as part of this PoC, 1491 outlier welds were evaluated, and, when classified as faulty, added to ZMON. Due to capacity limitations of the experts, only the majority of strong outliers and a selection of weak outliers were given for evaluation. However, the majority of the 25,000 welds remained unlabeled. To assess the performance of ABA-based classification, exploited here for the first time, faulty welds which were not offered for evaluation were identified from the ZMON condition log using the process illustrated in Figure 11. Through this process, the defect dataset was extended to include non-visual inspection sources, such as ultrasonic testing [82], and was not solely composed of ABA-defined outliers. The inclusion of these samples was crucial as the subsequent classification procedure relied on data which comprised all observed cases of welds, regardless of their ABA status.

The process of linking rail defects, of a general nature, to weld-specific defects was initiated by extracting all rail faults from the ZMON database for the portions of track evaluated as part of this PoC. These rail defects were then associated to the presence of a weld in their vicinity, allowing for a tolerance of 10 m to account for potential inaccuracy in the reported positions. The resulting matches were then visually verified by the authoring team using the VCUBE image system in order to minimize occurrence of wrong matches due to positioning uncertainties. This process resulted in labels for approximately 544 damaged welds out of the original set of 25,000 labels. In the absence of a label, a weld was assumed to be healthy for the classification process in Section 4.2, which was naturally a strong assumption, since certain welds may simply have failed to be picked up as defective.

### 4.2. Classification of Weld Condition

To further improve the aforementioned thresholds for expert feedback, the expert knowledge stemming from EVA and the ZMON logs was taken into account by considering three types of models.

The Bayesian Logistic Regression (BLR) model was compared against a Binary Choice (BC) approach, and the Random Forest (RF) model. As an input, the supervised classification process (see also Figure 1) used the condition labels of ZMON, enhanced with the expert evaluation, shown in Table 5, to provide superior classification thresholds on the basis of the ABA-derived indicators. Here, it is worth remembering that the longitudinal level indicator is itself an ABA-derived indicator through the process of integration.

The ABA features of Table 3 were computed for all samples in the dataset presented in Section 4.1.3. The naming convention of the essential indicators was $T1(T2\left({\mathrm{ABA}}_{C}^{M}\right))$, with the related letter entries explained in Table 6.

The dataset was divided into a training dataset and a test dataset, with 80% of the samples included in the former and the remaining 20% included in the latter. The models were trained on the processed essential indicators with the assigned condition labels. In some studies, data imbalance and overfitting is prevented by using data augmentation techniques; however, generating such augmented datasets requires special care since the augmented datasets are prone to bias [83]. For this reason, resampling techniques were used instead of generative approaches to account for the large imbalance between the number of healthy welds compared to defective instances. Certain models, such as the RF-based scheme, require the adjustment of the class weights to account for the over-represented category during the training procedure [84]. This was, here, achieved by weighing the minority class proportionally to its count in order to achieve balanced weights

(11) $$w_{j}=\frac{n}{c\cdot n_{j}}$$

where the weight $w_{j}$ for class *j* is weighed inversely proportionally to the ratio between the number of samples $n_{j}$ of class *j* and the total number of samples *n*. For the weld assessment problem, we assigned a binary condition label (“healthy” or “defective”) and, thus, the number of classes *c* is 2. This balancing was only applied during the training phase of the RF.

The model performance assessment metrics used here were accuracy, recall, precision, F1-score, and the Area Under the Receiver Operating Characteristic Curve (ROC–AUC). A brief description of these metrics is provided in [85]. The accuracy metric measures the ratio of correct predictions over the total number of evaluated instances. For imbalanced datasets, other metrics are generally preferred, as accuracy is sensitive to imbalance. The precision is defined as the ratio of the correctly classified positives (true defects), also referred to as true postives (TP), versus all classified positive instances (predicted defects), either correctly (TP) or incorrectly (false positives, FP). A low precision score indicates the presence of a high number of false positives, which can be an outcome of imbalanced class or untuned model hyperparameters. The recall, also referred to as the true positive rate (TPR), is calculated as the ratio between the number of correctly classified positive samples (TP) versus the total number of actually positive samples, which includes true positives (TP) and false negatives (FN). Both precision and recall offer metrics on the classification reliability in terms of predicting positives. Low recall rates result in lower safety due to an increased number of false negatives. The F1-score is defined as the harmonic mean of precision and recall, and is calculated using the following equation

(12) $$F1-\mathrm{score}=\frac{2(\mathrm{precision}\cdot \mathrm{recall})}{\mathrm{precision}+\mathrm{recall}}$$

The *F*1-score is adopted for assessing models with large class imbalance, as it assigns equal weight to both precision and recall. The Area Under the Receiver Operating Characteristic Curve (ROC–AUC) is another metric which is commonly used to assess the performance of binary classifiers, as it assesses the quality of the distinction between positive and negative classes. The ROC–AUC is calculated using the following equation

(13) ROC–AUC=∫(TPR(FPR))dFPR

where TPR stands for the true positive rate (or recall) and FPR denotes the false positive rate, defined as FPR=FP/(TN+FP). The ROC–AUC score can be misleading when the dataset is highly imbalanced and, thus, it is best used together with the precision, recall and F1-score for the assessment of the model performance.

The model selection proposed in Section 3.4 was now trained on the training dataset, which was composed of 80% of the samples of Table 5. One should note that the unlabeled condition and the non-defective samples from EVA were here both assumed to be healthy samples. In practice, as previously noted, there is uncertainty both in the expert labeling, as well as regarding the completeness and up-to-dateness of the ZMON database.

Table 7 illustrates the classification scores for the formulated models. The BC models offered lower scores than the BLR and RF classifiers, as their univariate nature did not enable them to capture more complex relations. The BLR model offered scores that were nearly as high as the RF classifier, capturing the linear relations of the indicators. The RF classifier offered the highest scores as this model can capture the non-linear relations between indicators. The BC classifier was used to evaluate the single features listed in Table 3, such as the maximum lateral acceleration *Y* measured over a distance of 3m around the weld, averaged across all measurement channels $\mu (\max_{3m}\left({\mathrm{ABA}}_{Y}^{RAW}\right))$. Table 7 furthermore shows that for the univariate BC models, lateral acceleration features performed better, while vertical acceleration features had slightly lower performance. The EVA for outlier detection could have, in hindsight, been performed on such an improved indicator. However, because organizing feedback rounds with many experts and asset managers from different regions was a complex task, expert assessment on the updated indicators will only be performed in future work. In addition, it was observed that increased ABA values in the 0.5 to 2 kHz range, for both the vertical and lateral direction, could indicate the presence of a defect. The single indicator BC models yielded F1-scores between 10% and 32% for the best performing single features. From the BC models, we observed that both vertical and lateral acceleration features yielded similar F1-scores.

**Figure 12:**
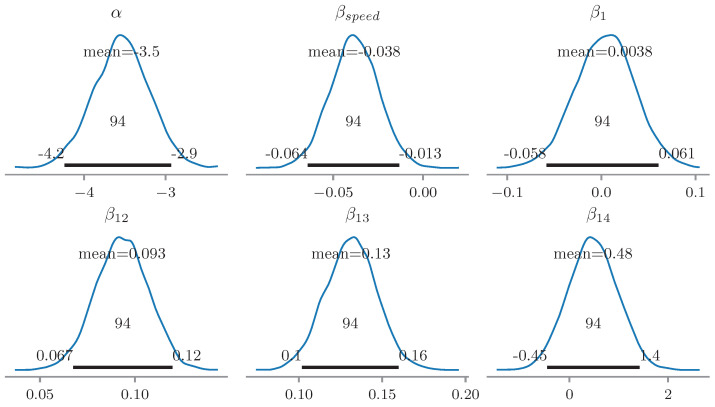
Posterior distributions of the BLR parameters α, $\beta_{speed}$, $\beta_{1}$, $\beta_{12}$, $\beta_{13}$, $\beta_{14}$. Mean and 94% HDI are reported in the plots. The essential indicators $x_{i}$ corresponding to the BLR parameters $\beta_{i}$ are listed in Table 4.

The BLR and RF models were trained using the 15 features yielding the highest F1-score in the univariate BC models and a cross-feature correlation of less than 0.8. Figure 12 illustrates the inferred posterior distributions for a selection of 6 parameters of the BLR model, inferred by MCMC inference with the samples collected after convergence of the chains. The essential indicators $x_{i}$ corresponding to the BLR parameters $\beta_{i}$ are summarized in Table 4. It was observed that certain inferred parameter distributions, such as $\beta_{1}$ and $\beta_{14}$, assigned high posterior probability to 0, suggesting low statistical significance. For example, $\beta_{14}$ corresponded to one of the longitudinal level D0 indicators, which further confirmed the limited value in geometric indications for welds, which are, indeed, nowadays mainly assessed visually and not in terms of geometry. Likewise, the other displayed variables considerably differed from the prior, and the 0 value was not contained in the 94% Highest Density Interval (HDI) [86], which supported their statistical significance.

The best BLR model yielded an F1-score of around 43%, while the best-performing RF model delivered an F1-score of around 48%. The models based on multiple indicators performed better than the ones using only a single feature. By further including the speed, which is an important operational parameter, as an input to the RF and the BLR models, the classification metrics were further improved. While the BLR delivered slightly lower metrics than the RF, it presented a reduced risk of over-fitting. Moreover, the BLR model additionally provided estimates of the predictive uncertainty.

**Figure 13:**
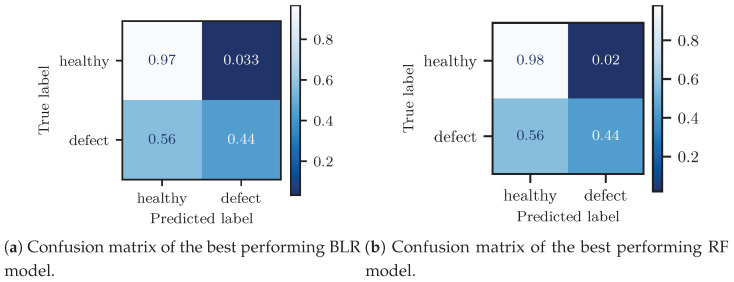
Confusion matrices for the BLR and RF classification models. The recall rate was around 44% and only 2% of the samples were mislabeled by the classifiers for the healthy scenario.

For all models, a trade-off always existed between recall and precision. When the recall increased, the precision decreased, resulting in a safer monitoring scheme, which, however, also delivered many false positives. To further illustrate this point, the confusion matrices for the best performing BLR and RF classifiers are shown in Figure 13a,b, respectively. While only about 2–3% false positive predictions occurred, the number of false negatives was up to 56%. One can observe in Figure 13 that the BLR and RF models had similar recall rates, but different precision, as the RF offered slightly less false positives. The decision threshold was set to maximize the F1-score. In practice, this decision threshold can be dynamically adjusted. For example, more ambiguous samples, such as weak outliers, could be considered for expert validation, but this would be associated with a higher false positive rate and a greater validation effort. Concerning the BLR model, the expert-in-the-loop approach could have been further extended to exploit the estimated predictive uncertainty, which offers a quantification of the confidence we may attribute to each judgment [87,88]. The predictive uncertainty could then be exploited for a second iteration of expert labeling on those samples associated with high uncertainty. The resulting reduced uncertainty in the labels would lead to subsequent improvement in the predictive models. While this extension was not considered in this PoC, for simplicity and to present a fair model comparison, it would certainly be useful to consider this for a practical real-world implementation, in order to fully exploit the BLR model capabilities.

**Figure 14:**
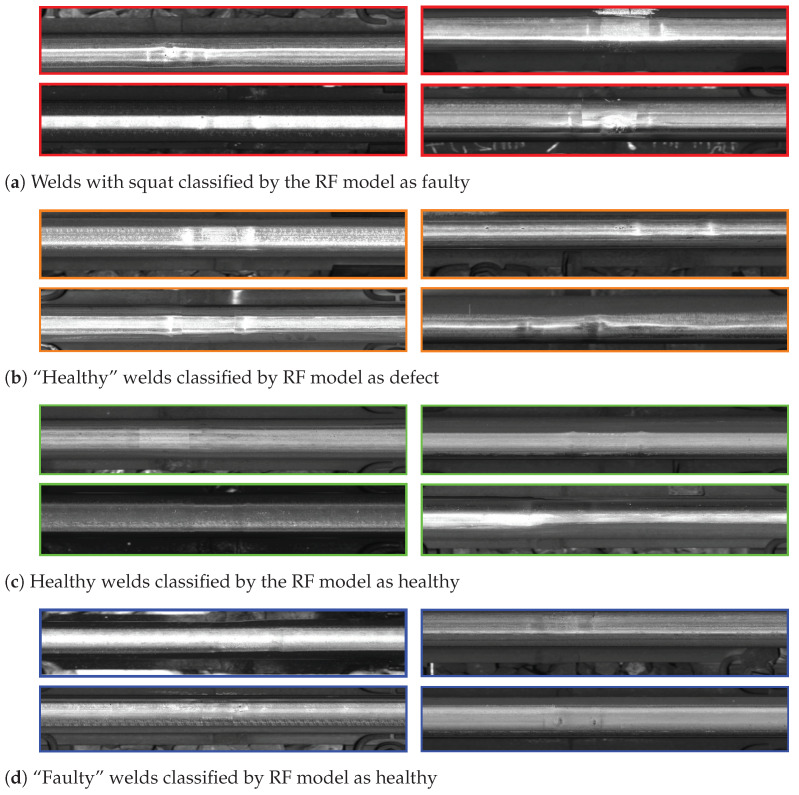
Challenges in the labeling quality of the dataset are showcased with images extracted from VCUBE of characteristic healthy and defective weld component classes for each classification scenario.

We noticed that the best performing RF model sometimes mistakenly classified healthy samples as defective and vice versa. Indeed, we noticed that the relatively low precision and recall could be attributed to the high uncertainty in the labels. Further examining the instances of correct and misclassified defects, we offer an illustration of some randomly selected instances for true positive (TP), false positive (FP), true negative (TN), and false negative (FN) instances in Figure 14. It is evident that these uncertainties were largely related to challenges in the quality of the dataset labeling. Many samples exhibiting acceleration and visual characteristics that would place them, respectively, in the healthy or defective category might effectively belong to a different condition category than the initially assumed one. Several reasons for these mislabeled samples exist:

1. The assumption that all welds not linked to a fault (from the ZMON database or the PoC expert-based evaluation) are healthy is inaccurate. Figure 14b illustrates that some samples assumed as healthy, but classified as defective (categorized as False positives), were indeed defective as they were not recorded in the ZMON database at the time of the inspection.
2. Additionally, errors can occur when matching the potentially outdated time and inaccurate location of defects recorded in the ZMON database with the weld observations. This can result in false negatives, where one can visually observe faults, as shown in Figure 14d.
3. Finally, additional uncertainty arises from expert judgment, which tends to vary considerably as each expert can reach a differing conclusion on the same sample.

Furthermore, experts often only generate new faults in the ZMON database for advanced damage conditions (see also Figure 14c), where the resulting maintenance is carried out within a prescribed time horizon. While this is a resource-efficient strategy, it is crucial to realize that accelerations are quite sensitive to moderate-intensity defects that lead to early alarms, as is shown in the next section. A binary classification is less suitable in such a scenario because it does not distinguish between different damage levels; this implies that damage severity and its progression need to be taken into account, which is only feasible under continuous tracking over time.

### 4.3. Continuous Tracking of Health Condition

The ABA-based classification of weld condition in Section 4.2 was performed on the basis of individual observations on welds. This work advocates adoption of an OBM paradigm, whose purpose is to deliver regular data collection from either diagnostic vehicles or appropriately instrumented in-service trains. In this way, tracking of the condition and the possible evolution of damage can be accomplished. By combining consecutive ABA measurements over time, the evolution of the rail infrastructure condition can be better estimated [7]. In this section, patterns and trends were identified with respect to the evolution of welds prior to reaching a damaged state, by analyzing the indicators derived from ABA over time. This information could lead to early detection of damage, and, in this way, facilitate the scheduling of timely maintenance activities and the improvement of rail infrastructure reliability.

**Figure 15:**
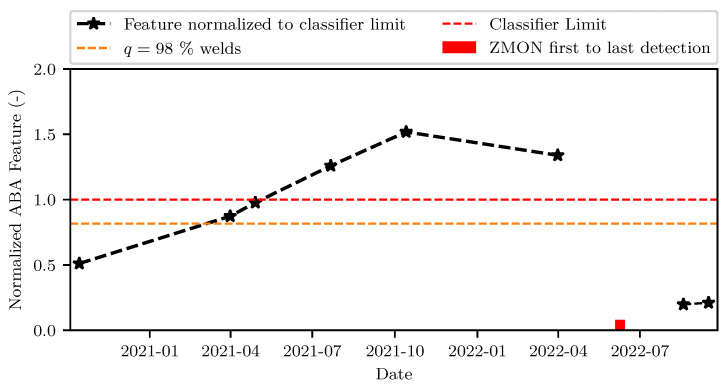
Time history of best performing feature of Section 4.2 normalized to the decision limit of the classifier. The ABA feature grew linearly until November, 2021. In April, 2022, rail grinding maintenance occurred on the track section, resulting in slightly lower ABA. The weld was labeled as faulty in June, 2022, by the experts and, subsequently, replaced in August, 2022.

Figure 15 plots the evolution of the ABA-derived indicator observed on a weld with a squat over time from October, 2020, until November, 2022. The indicator $\mu (\max_{2m}\left({\mathrm{ABA}}_{YZ}^{VS\;\mathrm{ABA}}\right))$ that was selected for this comparison was the one returning the highest F1-score for the BC model (see also Table 7), while simultaneously combining both vertical and lateral acceleration information. The ABA feature was normalized to the decision threshold γ defined in Equation (5). The normalized ABA feature grew linearly until November, 2021, for the weld of this case study. During the subsequent measurement in April, 2022, the ABA indicator decreased, due to rail grinding maintenance, which took place in March, 2022. The two thresholds highlighted in Figure 15 corresponded to the 98-th percentile which was applied during the initial expert evaluation round, and to the limit value for maximizing the F1-score during the classification process respectively. The first introduction of the weld of Figure 15 in the ZMON database occurted over a year after the highlighted thresholds were crossed showing the significant potential of using ABA as an early indicator of weld condition. Once the defect was identified, the weld was replaced within months, resulting in a recovery of low indicator values. In conclusion, the ABA-based indicator was able to track the true evolution of condition and deliver an indicator of real time damage progression.

**Figure 16:**
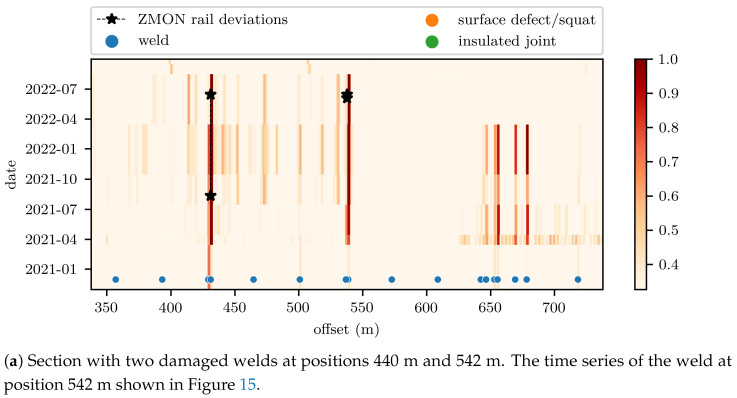

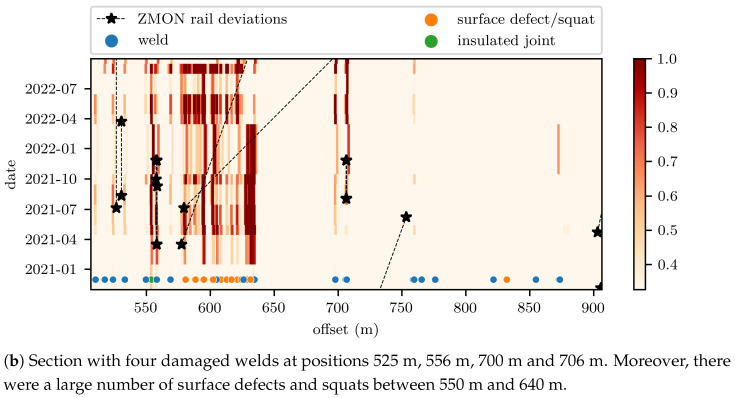
Heatmaps illustrating the spatial and temporal evolution of the ABA-based rail condition for two 400 m rail sections on different track segments. The detected welds, surface defects and insulated joints corresponded to unvalidated samples from the rail-head image-based detection and are marked with color bullets. The start and end point/time of the faults that are recorded in the ZMON database are shown as two stars linked by a dotted line. Locations with increased ABA indicators matched the welds which were recorded as faulty, or the sections with a high density of rail faults.

When considering larger scale infrastructure, it may often be more efficient to consider the assets over space and time, as a large number of welds exist on the network and tracking each one individually is resource intensive. The indicator of Figure 15 can be computed at any position on the track and visualized as time series plots, or as heatmaps. Asset managers are nowadays trained on interpreting heatmaps to analyze the variation of Track Quality Indicators (TQIs) over large track sections over time [89]. Figure 16a,b illustrates the heatmap for two rail sections of 400 m, where the rail condition indicator was normalized to the limit value for decision making defined in Section 4.2. The color scale of the heatmap reflects the increasing level of rail damage. The unvalidated welds, surface defects and insulated joints automatically detected by the VCUBE image-based detection are shown at the bottom of the plot. Increased indicator values are most commonly caused by insulated joints, faulty welds, surface defects or squats [51]. This is supported by Figure 16, as the increased indicator values lined up with these infrastructural elements. The start point/time and end point/time of the faults, recorded in the ZMON database, are shown as two stars linked by a dotted line, had lower space and time resolution, as they could be driven by other considerations. Figure 16a shows a section with two defect welds, including the one having the time series of the weld at position 542 m from Figure 15, while Figure 16b shows a section with several defective welds (at positions 525 m, 556 m, 700 m and 706 m). All the damaged welds showed clear growth of the indicator values over time. Beyond the application of ABA for detecting faulty welds only, these indicators characterize the rail surface roughness and, thus, they can be applied for all types of rail surface faults. For instance, a large quantity of surface defects occurred on the rail between position 550 m and 640 m in Figure 16b. In such cases, individual consideration of faults is of limited use, as it is most optimal to maintain the entire rail section at once.

In summary, the extraction of regular monitoring observations by means of specialized or in-service measurement vehicles equipped with ABAs bears potential for automating rail fault diagnostics. This can enhance predictive maintenance schemes by presenting asset managers with continual and spatially dense supervision of the rail condition over time.

## 5. Conclusions

In this work, we present a holistic framework for the automated detection of weld defects, by fusing a variety of observations, including on-site and visual inspections, automated diagnostic information extracted from monitoring vehicles and expert assessment. The scheme capitalizes on the availability of ABA information, extracted from accelerometer sensors featured on a diagnostic vehicle of the SBB. Extreme Value Analysis models were initially calibrated on various metrics stemming from the ABA measurements, in order to identify outlier welds in an unsupervised fashion on the basis of defined thresholds. The selected outliers were then passed onto actual field experts, in a first of its kind Proof-of-Concept project in collaboration with the SBB. The experts offered their feedback on rail-head images (VCUBE) that corresponded to the EVA-identified outliers. This novel application combined real world data with expert feedback and was executed in four evaluation rounds, carried out over a period of one year. Newly identified defects as part of this PoC were then entered into the condition monitoring database of the SBB, where they wewre fused with existing information from further evaluation processes.

The extended dataset was then used to develop an automated one-class classification scheme, whose purpose was to identify defective welds from the assimilated expert feedback. Three different methods were applied to this end: BC, RF classifiers and BLR. On the basis of the conducted analysis, it is possible to suggest a preferred measurement configuration for ABAs. We recommend the use of both vertical and lateral accelerations in the assessment procedure, as both are impacted by faults on welds. As such faults tend to induce an impulse-type of vibration response of the axle system, characterized by frequencies in the kilohertz range, this further motivates use of high frequency sensors. In terms of the performance of the suggested classification schemes, both the BLR and the RF models were trained on the same features, comprising the 15 features of the BC analysis that yielded the highest correlation to defect welds, while exhibiting less than 80% cross-feature correlation. The BLR model comes with the further advantage of delivering a prediction probability, which expresses the level of confidence we may attribute to the resulting labels.

These results indicate that component-specific evaluation can be delivered by combining asset type information with acceleration-based indicators and expert evaluation. Such an early detection of defects facilitated by acceleration-based indicators may improve the safety, efficiency, and cost effectiveness of both the inspection and maintenance process of rail welds in the future.

Finally, ABA measurements can detect faults much earlier, at their initiation, and, in this way, yield an estimate on damage severity. The continuous rating of weld condition over time, as opposed to the binary healthy/defective rating, further argues toward the importance of long-term monitoring schemes, which allow for tracking of condition over time. In an effort to demonstrate this, we presented examples which demonstrated that, prior to maintenance actions, significant growth of the ABA indicators was observed over time. This implied that emerging (early) faults were not caught by the experts, but were identified by the ABA and could feasibly be linked to a continuous indicator (rather than a categorical variable—label). Such ABA-derived indicators bear strong potential for effectuating early detection of faults and enabling a more granular and objective assessment of rail infrastructure condition.

While the proposed models are successful at classifying accelerations, further improvements can be obtained by some extensions to the current framework. An unsupervised approach using the Mahalanobis distance will be used in future work in order to allow assignment of labels beyond one-class classification, rather than on the the basis of a continuous scale, i.e., in terms of fault intensity [74]. The probabilistic framework enabled by the BLR can be extended by taking such a distance metric into account, while simultaneously incorporating the uncertainty in the expert labeling [90]. This work shows that such models improve the current paradigm of automated rail-head image-based inspection, but in the long-term pave the path for establishing OBM-based rail condition monitoring. The predictions of the proposed models can be incorporated as observations into a sequential decision-making framework to support optimal maintenance planning of railway assets [91].

## Figures and Tables

**Table 1:** Summary of the state-of-the-art of rail condition assessment with a focus on works exploiting ABA.

Proposed Approach	Research Findings	Limitations
Time-frequency analysis via Continuous Wavelet Transform (CWT)	Demonstrated identification of rail faults using the Scale-Averaged Wavelet Power of the CWT of ABA [33,34,35,36,37] or bogie measurements [38].	While the methodology can be extended to other components, these studies are limited to the assessment of squats.
Principal Component Analysis (PCA) on FFT coefficients.	Threshold for evaluating the condition of welds for the prioritization of inspection schedules [44].	Definition of linear weighting on the vehicle speed. The authors note that further studies are necessary to substantiate this indicator.
Wavelet Packet Decomposition (WPD) and Adaptive Synchro-squeezed Short Time Fourier Transform.	The authors identify the 300∼800 Hz frequency range to be indicative for poorly welded joints [45].	Empirical definition of a fixed damage threshold.
Hilbert-Huang Transform (HHT).	Rail joints were detected as impact points and outliers in the ABA signal with the HHT [46]	The methodology does not differentiate between components and only is demonstrated on a couple of samples.
Deep Learning architectures.	Classification of rail faults using Random Forests, Support Vector Machine, Artificial Neural Networks or Convolutional Neural Networks [40,50,51,52,53].	Requires large training datasets, especially when using acceleration time series instead of features as an input. Complex models require special care since they have a higher risk of overfitting.
Model & simulation based approaches.	Diagnostic thresholds were defined for faulty welds on the basis of ABA or force response simulations [30,31,36,56].	No generalization to generic geometries and track types; simulation may not fully reflect complex site conditions and noisy data.
Current Work: Fusion of ABA-derived indicator with expert feedback.	Statistical methods applied on essential indicators for the identification of weld condition [55].	Uncertainties in the expert assessment cause noise in the input labels for classification algorithms.

**Table 2:** Meaning of the $D_{A\;S}$ naming convention of the sensors.

Letter	Explanation	Possible Entries
*D*	direction	*Y* for lateral, *Z* for vertical
*A*	axle number	1 to 4, starting from the front (leading) axle
*S*	vehicle side	1 for right, 2 for left (w.r.t vehicle’s top view)

**Table 3:** Fundamental quantities used for the feature extraction process.

Feature	Quantity	Signal Length
RAW ABA	Raw accelerations	2 m, 3 m
VS ABA	Vector sum of Y and Z axes	2 m
BP ABA	Band Pass filtered accelerations	3 m
STFT	Short Time Fourier Transform	2 m
DWT	Discrete Wavelet Transform	0.625 s
D0 & D1	Longitudinal level and lateral displacement	5 m, 20 m

**Table 4:** Summary of the 15 indicators with the highest F1-score and a cross correlation of under 80% that were input to the BLR and RF models.

Index	Feature
$x_{1}$	$\max(\max_{2m}\left({\mathrm{ABA}}_{Z}^{STFT\;2800Hz}\right))$
$x_{2}$	$\max(\max_{2m}\left({\mathrm{ABA}}_{Z}^{STFT\;5800Hz}\right))$
$x_{3}$	$\max(\max_{2m}\left({\mathrm{ABA}}_{Z}^{STFT\;3000Hz}\right))$
$x_{4}$	$\mu (\max_{2m}\left({\mathrm{ABA}}_{Y}^{STFT\;1400Hz}\right))$
$x_{5}$	$\max(\max_{2m}\left({\mathrm{ABA}}_{Z}^{STFT\;5400Hz}\right))$
$x_{6}$	$\max(\max_{2m}\left({\mathrm{ABA}}_{Z}^{STFT\;400Hz}\right))$
$x_{7}$	$\max(\max_{0.625s}\left({\mathrm{ABA}}_{Z}^{DWT\;Haar\;cD5}\right))$
$x_{8}$	$\mu (\max_{3m}\left({\mathrm{ABA}}_{Y}^{BP\;0.2-0.5kHz}\right))$
$x_{9}$	$\mu (\max_{3m}\left({\mathrm{ABA}}_{Z}^{BP\;0.1-11kHz}\right))$
$x_{10}$	$\max(\max_{0.625s}\left({\mathrm{ABA}}_{Y}^{DWT\;Haar\;cD6}\right))$
$x_{11}$	$\max(\max_{2m}\left({\mathrm{ABA}}_{Z}^{STFT\;1400Hz}\right))$
$x_{12}$	$\max(\max_{2m}\left({\mathrm{ABA}}_{Y}^{STFT\;1400Hz}\right))$
$x_{13}$	$\max(\max_{2m}\left({\mathrm{ABA}}_{Z}^{STFT\;800Hz}\right))$
$x_{14}$	$\min(\min_{3m}\left({\mathrm{ABA}}_{Z}^{Long.\;level\;D0}\right))$
$x_{15}$	$\max(\max_{3m}\left({\mathrm{ABA}}_{Z}^{Long.\;level\;D0}\right))$
$x_{speed}$	vehicle velocity

**Table 5:** Number of welds in the database after a monitoring period of one year for the selected portion of tracks on the SBB network.

Condition	No Defect	Defect		No Defect
Label Source	EL-EVA ^1^	EL-EVA ^1^	ZMON ^2^	Unlabeled Condition ^3^
Low Outliers	0	0	368	19668
Weak Outlier	656	42	141	2242
Strong Outlier	710	83	54	285

^1^ EL-EVA stands for Expert Labels from the Extreme Value Analysis. ^2^ Defect welds from the condition monitoring database ZMON originate from the standard manual and automated track inspection process, or from the ultrasonic assessment of welds. ^3^ The samples with unlabeled condition correspond to welds that were not submitted for expert evaluation or assigned an existing defect within ZMON and are, here, assumed to be healthy.

**Table 6:** Meaning of the $T1(T2\left({\mathrm{ABA}}_{C}^{M}\right))$ naming convention of the essential indicators.

Letter	Explanation	Possible Entries
T1	Summary statistic computed over all (*Y*/*Z*) sensor channels	mean μ, standard deviation σ, min, max…
T2	Summary statistic computed over time series where the subscript denotes the window size	mean μ, standard deviation σ, min, max…
*M*	Applied method and parameters	STFT, Longitudinal Level D0, DWT…
*C*	Sensor channel	*Z* for vertical, *Y* for lateral

**Table 7:** Defect weld classification scores on the ZMON test dataset for the best performing features of each feature type of the Binary Choice (BC), as well as the Random Forest (RF) and Bayesian Logistic Regression (BLR) models that yielded the best F1-scores (see also the feature naming convention in Table 6).

Classifier	Features	F1	Roc-Auc	Accuracy	Recall	Precision
BC	$\mu (\min_{3m}\left({\mathrm{ABA}}_{Z}^{Long.\;level\;D0}\right))$	0.109	0.553	0.290	0.846	0.058
BC	$\mu (\max_{3m}\left({\mathrm{ABA}}_{Z}^{Long.\;level\;D0}\right))$	0.111	0.555	0.371	0.761	0.060
BC	$\min(\min_{3m}\left({\mathrm{ABA}}_{Z}^{Long.\;level\;D0}\right))$	0.116	0.542	0.799	0.256	0.075
BC	$\max(\max_{3m}\left({\mathrm{ABA}}_{Z}^{Long.\;level\;D0}\right))$	0.119	0.543	0.822	0.233	0.079
BC	$\mu (\sigma_{3m}\left({\mathrm{ABA}}_{Y}^{RAW}\right))$	0.250	0.610	0.921	0.265	0.236
BC	$\max(\max_{0.625s}\left({\mathrm{ABA}}_{Y}^{DWT\;Haar\;cD2}\right))$	0.259	0.647	0.893	0.373	0.198
BC	$\mu (\max_{0.625s}\left({\mathrm{ABA}}_{Y}^{DWT\;Haar\;cD3}\right))$	0.263	0.634	0.908	0.329	0.219
BC	$\mu (\max_{0.625s}\left({\mathrm{ABA}}_{Y}^{DWT\;Haar\;cD6}\right))$	0.264	0.645	0.898	0.364	0.207
BC	$\mu (\max_{0.625s}\left({\mathrm{ABA}}_{Y}^{DWT\;Haar\;cD2}\right))$	0.270	0.635	0.912	0.327	0.230
BC	$max(\max_{3m}\left({\mathrm{ABA}}_{Y}^{RAW}\right))$	0.293	0.668	0.902	0.408	0.229
BC	$\mu (\max_{3m}\left({\mathrm{ABA}}_{Y}^{RAW}\right))$	0.307	0.641	0.927	0.322	0.293
BC	$\max(\max_{2m}\left({\mathrm{ABA}}_{Y}^{STFT\;400Hz}\right))$	0.307	0.654	0.919	0.360	0.268
BC	$\mu (\max_{3m}\left({\mathrm{ABA}}_{Y}^{BP\;0.8-2kHz}\right))$	0.308	0.638	0.930	0.313	0.303
BC	$\mu (\max_{2m}\left({\mathrm{ABA}}_{YZ}^{VS\;\mathrm{ABA}}\right))$	0.315	0.650	0.925	0.344	0.291
BC	$\max(\max_{2m}\left({\mathrm{ABA}}_{Z}^{STFT\;800Hz}\right))$	0.323	0.655	0.926	0.355	0.295
BLR	15 indicators with highest F1-score ^1^ and cross-feature correlation under 0.8	0.422	0.696	0.937	0.426	0.417
BLR	15 indicators with highest F1-score ^1^ and cross-feature correlation under 0.8 & speed	0.431	0.701	0.938	0.432	0.427
RF	15 indicators with highest F1-score ^1^ and cross-feature correlation under 0.8	0.479	0.711	0.948	0.446	0.517
RF	15 indicators with highest F1-score ^1^ and cross-feature correlation under 0.8 & speed	0.486	0.708	0.950	0.436	0.550

^1^ The *F*1-scores of the indicators were defined with the univariate BC model.

## Data Availability

Not applicable.

## Abbreviations

The following abbreviations are used in this manuscript:

ABA	Axle Box Accelerations
BC	Binary Choice model
BLR	Bayesian Logistic Regression model
CWT	Continuous Wavelet Transform
DWT	Discrete Wavelet Transform
EVA	Extreme Value Analysis
gDFZ	Diagnostic Monitoring Vehicle of the SBB
OBM	On Board Monitoring
PoC	Proof-of-Concept
STFT	Short Time Fourier Transform
RF	Random Forest model
SBB	Swiss Federal Railways
*VCUBE*	Rail-Head Imaging System on the gDFZ
ZMON	Database for Condition Monitoring & Logging
